# The rising tide of rhegmatogenous retinal detachment in Germany: a nationwide analysis of the incidence, from 2005 to 2021

**DOI:** 10.1007/s00417-024-06392-2

**Published:** 2024-03-11

**Authors:** Ahmad Samir Alfaar, Peter Wiedemann, Matus Rehak, Armin Wolf

**Affiliations:** 1 Ophthalmology Department, Ulm University Hospital, Ulm, Germany; 2 Medical Neuroscience Program, Charité – Universitätsmedizin Berlin, Berlin, Germany; 3 St. Paul Eye Unit, Royal Liverpool University Hospital, Liverpool, UK; 4 Ophthalmology Department, Leipzig University Hospital, Leipzig, Germany; 5 Ophthalmology Department, Giessen University Hospital, Giessen, Germany; 6 Ophthalmology Department, Medical University of Innsbruck, Innsbruck, Austria

**Keywords:** Retinal detachment, Incidence, Trends, Risk factors, Epidemiology, Sex, Age

## Abstract

**Purpose:**

This nationwide study aims to delineate the incidence and trends of rhegmatogenous retinal detachment (rRD) in Germany across 17 years (2005–2021).

**Methods:**

We conducted a retrospective cohort study using data from the German Federal Statistics Office and the Institute for the Hospital Remuneration System (InEK). The dataset includes approximately 19 million annual inpatient admissions annually. Retinal detachment was identified through ICD-10 code H33.0. Adjusted incidence rates were estimated after excluding reoperation cases. We used R Statistical Software to calculate estimates to 2021, and Tableau for visualisation.

**Results:**

From 2005 to 2021, Germany reported 332,650 rRD cases, with males consistently more affected. Adjusted incidence rose from 15.6 per 100,000 in 2005 to 24.8 in 2021. Variable annual percentage changes in incidence were noted, averaging 4.0% for males and 2.6% for females. The annual mean age of affected individuals ranged from 60.2 to 62 years, with a median age between 62 and 63, suggesting increasing diagnoses at younger ages. Hospital stays declined from 6 to 3.3 days, and higher management rates were observed in Saarland and Rhineland-Palatine.

**Conclusion:**

The study confirms an increasing incidence of rRD in Germany from 2005 to 2021, particularly among males. These findings call for further research to investigate the underlying causes. Collaboration among healthcare professionals, researchers, and policymakers is essential for effective management and improved visual outcomes.

**Supplementary Information:**

The online version contains supplementary material available at 10.1007/s00417-024-06392-2.

## Introduction

Rhegmatogenous retinal detachment (rRD) is a critical ocular emergency potentially precipitating severe visual impairment or even irreversible blindness if not addressed with immediate and appropriate intervention [1, 2]. The rhegmatogenous type, provoked by retinal breaks that allow fluid migration from the vitreous to the subretinal space, demands urgent surgical intervention within a hospital environment. Despite the acute nature of this condition, the discourse surrounding its incidence and associated risk factors remains in a state of flux, with ongoing research endeavours seeking to delineate these aspects more clearly [3]. The level of our understanding in this field directly shapes the development of potent preventative strategies and the innovation of targeted therapeutic avenues. In light of this, our study is committed to fortifying the existing body of knowledge by scrutinising a substantial, nationwide dataset of rRD cases in Germany, thereby addressing the current gaps in the literature.

While numerous studies have reported the incidence of rRD globally, the existing corpus of literature exhibits several shortcomings[4, 5]. Initial investigations, including the notable Mainz study, have given valuable insights into the incidence of rRD within specific populations and age groups in Germany [6]. However, these studies have often been constrained by their exclusion of specific age demographics, reliance on restricted sample sizes, or the absence of comprehensive data encompassing various rRD subtypes. Moreover, these research efforts have largely been unable to quantify the influence of evolving ocular surgical techniques and the increasing prevalence of myopia on the incidence of rRD, with only a handful addressing the temporal and geographical variations in incidence [7, 8].

Our study aims to elucidate the incidence of rhegmatogenous rRD in Germany on a national scale and across a comprehensive range of age groups while delineating the patterns associated with different rRD subtypes. Through this approach, we aspire to cultivate a renewed understanding of the epidemiology of rRD in Germany. This endeavour will facilitate clinicians and researchers in identifying populations at elevated risk with greater accuracy, evaluating the efficacy of current preventative and treatment strategies, and crafting targeted interventions to mitigate the impact of rRD on patients and healthcare infrastructures. In doing so, we seek to fulfil the pressing need for updated epidemiological data, particularly in the face of advancements in ocular surgical practices and the rising tide of myopia.

## Methods

### Study design and setting

We conducted a comprehensive, nationwide, retrospective cohort study to scrutinise the incidence and temporal variations of rRD in Germany. The study meticulously analysed data from January 1 to December 31 of each respective year, culminating in 2019, with an extended analysis encompassing the period of 2019 to 2021.

### Data source and collection

Due to the German refunding system, rRD is not treated in office settings.

Therefore, the primary data repositories for this study were the hospitals admission data from the German Federal Statistics Office (Statistisches Bundesamt) and the Institut für das Entgeltsystem im Krankenhaus GmbH (INEK). In this study, we adhered to the InEK GmbH's usage terms by employing the InEK DatenBrowser solely for research. The dataset, a robust compilation, encapsulated approximately 19 million cases of inpatient admissions annually. Case identification was facilitated by using the ICD-10 code H33.0, earmarked for rRD. Data about serous and tractional rRD cases were gathered and excluded from the incidence analysis to secure an accurate analysis.

### Ethical considerations

To adhere to stringent ethical guidelines, the data, initially anonymised by the respective providers and subsequently made publicly accessible, ensured the absolute non-disclosure of patient identities. Furthermore, data groups documenting fewer than four patient frequencies were systematically censored, categorising our study as non-human subject research and obviating the necessity for Institutional Review Board (IRB) approval.

### Participants

The study encompassed individuals admitted due to rRD within the stipulated timeframe, devoid of restrictions on age or gender demographics.

### Variables and data measurement

The pivotal outcome variable under investigation was the incidence of rRD. Ancillary variables encompassed patient demographics, duration of hospitalisation, and the frequency of reoperations (Code 9-983).

### Statistical analysis

The analytical process was facilitated by deploying Microsoft Excel, Google Sheets, and Tableau Software. We embarked on calculating crude incidence rates per 100,000 individuals, with demographic parameters sourced from the Robert Koch Institute.

### Bias and sample size

Potential biases, including selection and information bias, were mitigated through a comprehensive data collection process and the inclusion of a substantial sample size. To derive a more accurate estimate of the incidence rate for new cases of rRD, we adjusted the total number of admissions by excluding cases related to reoperations. This adjusted rate aims to better represent the incidence rate within the spectrum of rates calculated before and after accounting for reoperation admissions. We employed reoperation data from 2019 to 2021 to construct predictive model that extrapolate reoperation rates back to 2005 assuming a linear trend, informed by the observed pattern of increase over the 3 years from 2019 to 2021. Subsequently, we calculated the incidence rates based on these retrospective regressions extending back to 2005.

## Results

### General trends

Over the span of seventeen years (2005–2021), there were a total of 332,650 reported cases of rRD in Germany, with males accounting for a larger proportion of cases than females across all years. The data from Statistisches Bundesamt and InEK showed a continuous increase in the frequency of rRD until 2019, with a decline in later years. The datasets from both institutes exhibited high congruence for the fiscal year 2019, thereby validating the continued utilisation of InEK data through the conclusion of 2021.

### Sex disparities

Males consistently represented a larger proportion of rRD cases, increasing from 59.2% in 2005 to a peak of 64.6% in 2020. Correspondingly, the female percentage ranged from a high of 40.9% in 2005 to a low of 35.4% in 2020.

### Incidence rates

**Fig. 1:**
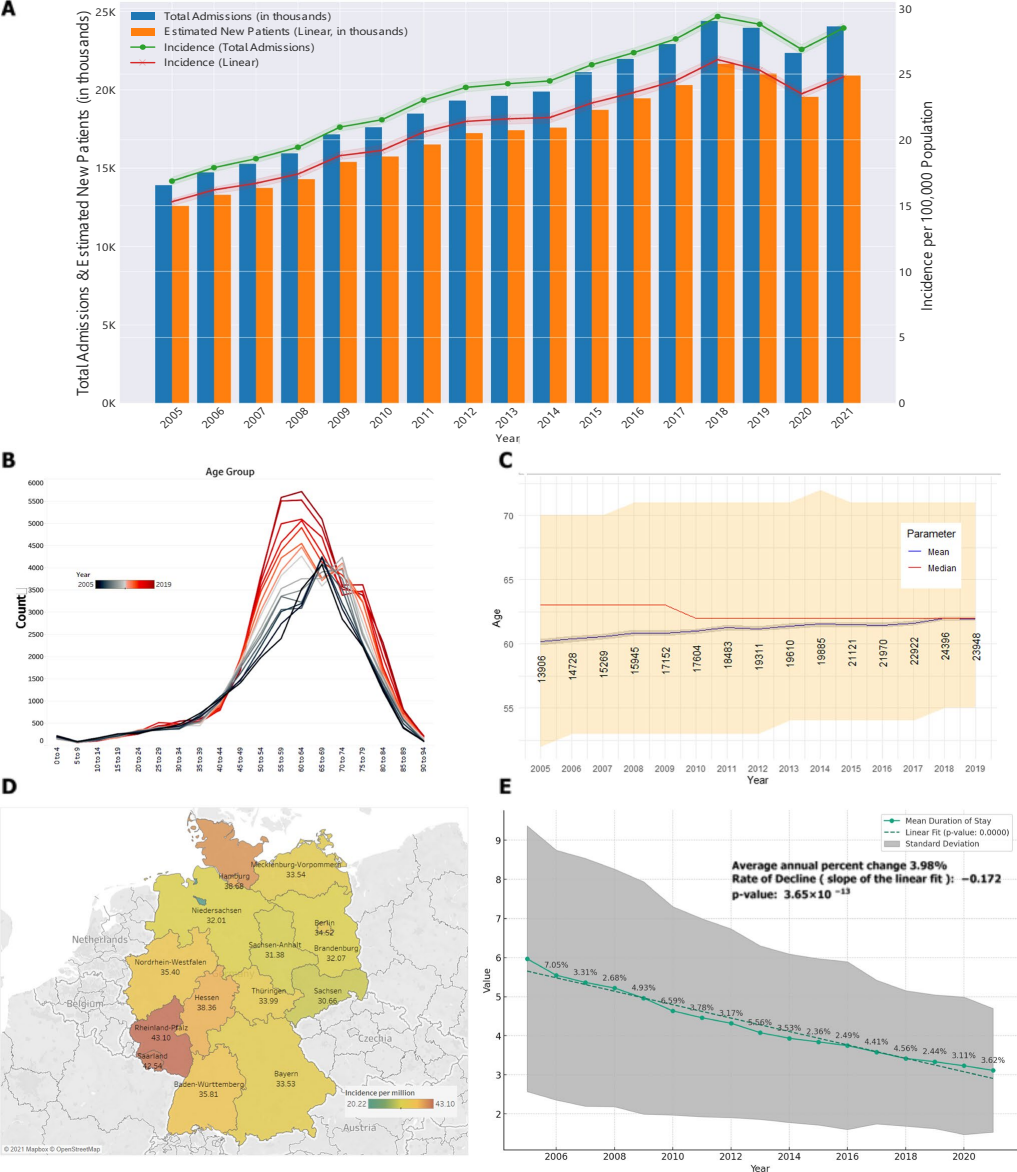
**A**. Incidence of rhegmatogenous retinal detachment between 2005 and 2021 (admissions and estimated de novo incidence). **B**. number of admissions with retinal detachment per age group. **C**. Relation between mean and median age at presentation of retinal detachment. **D**. Distribution per state (per 100,000 persons). **E**. Postoperative hospital stay. Sources: A. and E: DESTATIS, InEK and modeling. B, C, D: DESTATIS. Acronyms: ReOp: Re-operations. Note: Only Fig. 1A was adjusted for re-operations

The incidence rates (represented as admission rates) of rRD in both males and females increased over the years. For males, the incidence increased from 20.4 per 100,000 (95% CI: 20 − 20.8) in 2005 to a peak of 38.3 per 100,000 (95% CI: 37.7–38.8) in 2018 (Table 1). For females, the admission increased from 13.5 per 100,000 (95% CI: 13.1 − 13.8) in 2005 to a peak of 20.8 per 100,000 (95% CI: 20.3 − 21.2) in 2018. When we exclude the reoperations, de novo incidence rates increased from 15.3 (95% CI: 15.0 − 15.5) per 100,000 in 2005 (considering linear reoperation modelled rates) to 24.8 in 2021 (95% CI: 24.5 − 25.1) (Fig. 1, Supplementary Table 1).

**Table 1:** Frequency of admission, admission rate, age, and data source

Year	Frequency of admissions			Percent		Admission rate per 100,000 (95%CI)			Percent change			Age		Source
Year	Male	Female	Total	Male	Female	Male	Female	Total	Male	Female	Total	Mean (95%CI)	Median (IQR)	
2005	8226	5680	13,906	59.2%	40.9%	20.4 (20–20.8)	13.5 (13.1–13.8)	16.9 (16.6–17.1)				60.2 (59.9–60.4)	63 (52–70)	SBA
2006	8868	5860	14,728	60.2%	39.8%	22 (21.5–22.5)	13.9 (13.6–14.3)	17.9 (17.6–18.2)	7.9%	3.4%	6.1%	60.4 (60.1–60.6)	63 (53–70)	SBA
2007	9198	6071	15,269	60.2%	39.8%	22.8 (22.4–23.3)	14.5 (14.1–14.8)	18.6 (18.3–18.9)	3.8%	3.8%	3.8%	60.6 (60.3–60.8)	63 (53–70)	SBA
2008	9601	6344	15,945	60.2%	39.8%	23.9 (23.4–24.4)	15.2 (14.8–15.5)	19.4 (19.1–19.7)	4.6%	4.8%	4.7%	60.9 (60.6–61.1)	63 (53–71)	SBA
2009	10,587	6565	17,152	61.7%	38.3%	26.4 (25.9–26.9)	15.7 (15.4–16.1)	21 (20.7–21.3)	10.5%	3.8%	7.8%	60.8 (60.6–61.1)	63 (53–71)	SBA
2010	10,759	6845	17,604	61.1%	38.9%	26.8 (26.3–27.3)	16.4 (16–16.8)	21.5 (21.2–21.9)	1.6%	4.4%	2.7%	61 (60.8–61.2)	62 (53–71)	SBA
2011	11,674	6809	18,483	63.2%	36.8%	29.8 (29.2–30.3)	16.6 (16.2–17)	23 (22.7–23.3)	10.9%	0.8%	6.9%	61.3 (61.1–61.5)	62 (53–71)	SBA
2012	11,978	7333	19,311	62.0%	38.0%	30.4 (29.9–31)	17.8 (17.4–18.2)	24 (23.6–24.3)	2.2%	7.6%	4.2%	61.2 (61–61.4)	62 (53–71)	SBA
2013	12,258	7352	19,610	62.5%	37.5%	31 (30.4–31.5)	17.8 (17.4–18.2)	24.3 (23.9–24.6)	1.9%	0.1%	1.2%	61.4 (61.2–61.6)	62 (54–71)	SBA
2014	12,547	7338	19,885	63.1%	36.9%	31.5 (30.9–32)	17.7 (17.3–18.1)	24.5 (24.1–24.8)	1.6%	− 0.6%	0.9%	61.5 (61.3–61.7)	62 (54–72)	SBA
2015	13,461	7660	21,121	63.7%	36.3%	33.2 (32.7–33.8)	18.4 (18–18.8)	25.7 (25.4–26)	5.5%	3.6%	5.0%	61.5 (61.3–61.6)	62 (54–71)	SBA
2016	13,988	7982	21,970	63.7%	36.3%	34.4 (33.8–34.9)	19.1 (18.7–19.5)	26.6 (26.3–27)	3.4%	3.8%	3.6%	61.4 (61.3–61.6)	62 (54–71)	SBA
2017	14,589	8333	22,922	63.7%	36.4%	35.7 (35.1–36.3)	19.9 (19.4–20.3)	27.7 (27.3–28)	3.9%	4.1%	4.0%	61.6 (61.4–61.8)	62 (54–71)	SBA
2018	15,670	8726	24,396	64.2%	35.8%	38.3 (37.7–38.8)	20.8 (20.3–21.2)	29.4 (29–29.8)	7.1%	4.5%	6.1%	62 (61.8–62.1)	62 (55–71)	SBA
2019	15,410	8538	23,948	64.4%	35.7%	37.6 (37–38.1)	20.3 (19.8–20.7)	28.8 (28.4–29.2)	− 1.8%	− 2.3%	− 2.0%	61.9 (61.7–62.1)	62 (55–71)	SBA
2019	15,419	8543	23,962	64.3%	35.7%	37.6 (37–38.2)	20.3 (19.8–20.7)	28.8 (28.4–29.2)						InEK
2020	14,431	7924	22,355	64.6%	35.4%	35.2 (34.6–35.7)	18.8 (18.4–19.2)	26.9 (26.5–27.2)	− 6.4%	− 7.2%	− 6.7%			InEK
2021	15,486	8559	24,045	64.4%	35.6%	37.7 (37.1–38.3)	20.3 (19.9–20.7)	28.9 (28.5–29.3)	7.2%	7.9%	7.5%			InEK
Total	208,731	123,919	332,650				Average annual percent change		4.0%	2.6%	3.5%			

*SBA* Statistisches Bundesamt, *InEK* Institut für das Entgeltsystem im Krankenhaus, *CI* confidence interval

### Annual percent change in incidence rates

The incidence rates for rRD saw variable annual per cent changes from 2006 to 2021. For males, the highest annual per cent change was recorded in 2009 (10.5%), while for females, the highest change was recorded in 2012 (7.6%). On average, males had an annual per cent change of 4.0%, while females had a lower annual per cent change of 2.6% (Fig.1.E).

### Age distribution

Across the period, the mean age for individuals with rRD ranged from a low of 60.2 (95% CI: 59.9–60.4) in 2005 to a high of 62 (95% CI: 61.8–62.1) in 2018. The median age showed a relatively stable trend, fluctuating between 63 (IQR: 52–70) and 62 (IQR: 55–71) throughout the years (Fig. 1). The latter showed that more patients are diagnosed with RD at younger ages, recently.

### Hospital stay and distribution

Mean hospital stays declined from 6 days in 2005 to 3.1 days in 2021. The hospitals in the states of Saarland and Rhineland-Palatinate handled more patients per capita, while Bremen (and Saxony) showed the lowest rates.

## Discussion

RRD represents a critical medical emergency necessitating immediate surgical intervention to prevent severe visual impairment. While larger metanalyses demonstrate a large geographical variation, globally, the incidence of this condition has been on an upward trajectory, a trend confirmed by numerous studies conducted in various countries [5, 9]. Our research in Germany substantiates this escalating pattern, delineating a steady and pronounced increase in cases from 2005 to 2018.

In a comprehensive study conducted in Kumamoto, Japan, Ideta et al. in 1995 reported an annual incidence rate of 10.4 cases per 100,000 individuals, noting a significant correlation with lattice degeneration and macular holes contributing to higher detachment rates [10]. Similarly, a study spearheaded by Wong in 1999 scrutinised the incidence in Singapore, revealing a demographic predisposition wherein Chinese individuals exhibited the highest incidence rates, followed by Malays and Indians [11]. Furthermore, the study highlighted a heightened risk among males compared to females. In nearby countries and timing, Poland showed an incidence of 13.7 per 100,000 while France reported an incidence of 21.97 per 100,000 population between 2010 and 2016 [12, 13]. Adding to this body of research, Mitry’s 2010 investigation in Scotland documented an annual incidence rate of 12.05 per 100,000 population, emphasising a pronounced incidence in males and identifying a robust association between higher levels of affluence and increased incidence rates [14]. Like our study, the incidence of rRD in Scotland has steadily increased from 9.36 per 100,000 in 1987 to 13.61 per 100,000 in 2006; similarly, the French study showed an increase in the incidence until 2015.

Myopia, or nearsightedness, is a significant risk factor for rRD [15, 16]. The elongation of the eye in myopia can lead to the development of retinal tears, increasing the risk of detachment. The rising incidence of myopia globally, particularly in Asian countries, may contribute to increasing rRD [16–18]. Our findings support this association, as the incidence rates of rRD in Germany have increased during the study period.

Other factors associated with an increased risk of rRD include previous cataract surgery, ocular trauma, and age [2, 19, 20]. These factors may contribute to the development of retinal tears and subsequent detachment. Data from the IRIS registry indicates that at a younger age, the risk for rRD after cataract surgery is increased, along with other risk factors such as myopia, male sex, and others [21]. Therefore, a potential explanation for the observed increase in rRDs could be a trend to perform cataract surgery at earlier stages.

However, the trend of increasing rRD incidences is also observed in other countries with presumably constant age of patients at the time of cataract surgery.

Our study did not directly examine the impact of cataract surgery and other factors; however, their known associations with rRD support the need for further investigation and consideration in preventive strategies.

Our study also observed a higher incidence of rRD among males, consistent with the existing literature. Sex differences have been observed previously in the incidence of rRD [22]. Additionally, the increase of incident rates is constantly higher in the male population, indicating that the difference in incidences of rRD between the sexes will grow in future. Apart from lifestyle differences, hormonal factors may play a role. Oestrogen and progesterone receptors have been found in the eye, suggesting a potential hormonal influence on rRD. Further research is needed to elucidate the specific hormonal mechanisms and their contribution to sex disparities in rRD incidence [23].

The increasing incidence of rRD in Germany may be attributed to various factors. Age is a significant risk factor for rRD, and the ageing population could contribute to the rising incidence rates [24]. Age-related changes in the vitreous, such as posterior vitreous detachment and vitreoretinal traction, can predispose individuals to retinal tears and detachment [2, 7]. Our findings align with these observations, as the mean age of individuals with rRD in Germany increased over the study period, suggesting an age-related risk.

While rRD in children is relatively rare, it can occur in specific conditions such as Stickler syndrome and giant retinal tears [25, 26]. Our study showed a higher incidence rate in old populations; however, recognising specific conditions associated with paediatric rRD highlights the importance of targeted screening and management.

Migration patterns can have a substantial impact on healthcare metrics. The influx of younger populations into Germany, particularly around 2015, may have influenced the age-specific incidence rates of rRD. Younger populations may present with different risk profiles, such as fewer incidences of age-related rRD but potentially higher rates related to trauma or myopia.

The length of hospital stays for rRD in Germany has declined over the years. This reduction in hospital stays may be attributed to advancements in surgical techniques, improvements in postoperative care or changes in the refunding system. Modern surgical techniques, such as small-gauge pars plana vitrectomy, have contributed to shorter hospital stays for rRD patients [27]. These techniques have allowed for more efficient and minimally invasive procedures, resulting in faster recovery and shorter hospital stays. Additionally, improvements in postoperative care and management may have played a role in the decreased length of hospital stays. Enhanced pain management strategies, early mobilisation, and optimised discharge planning have all contributed to shorter hospital stays for rRD patients.

The shorter hospital stays for rRD in Germany also align with other countries’ trends. Studies conducted in various countries have reported similar findings, with decreased hospital stay length for rRD patients [28].

The reduction in hospital stays for rRD is beneficial for both patients and healthcare systems. Shorter hospital stays can lead to cost savings, improved patient satisfaction, and more efficient use of healthcare resources. Such a reduction may also be influenced by the insurance companies’ payment system, which tends to limit the reimbursement to specific budgets per diagnosis group.

It is important to note that the length of hospital stays may vary depending on the severity of the rRD, the surgical technique used, and individual patient factors. Therefore, the average length of hospital stays should be interpreted in the context of these factors.

The limited availability of specialised retinal surgeries, even in university hospitals, can affect both the recorded incidence and the outcomes of rRD. Reduced service availability, especially on weekends and holidays, can lead to delays in diagnosis and treatment, affecting the reported incidence rates and possibly exacerbating the condition’s severity. The decrease in incidence observed since 2020 is likely linked to the COVID-19 pandemic. Caution is advised when interpreting regression analyses affected by this period due to pandemic-related disruptions. A resurgence in incidence as conditions normalize should not be overlooked, warranting continuous monitoring.

Despite the valuable insights provided by our study, certain limitations should be acknowledged. Firstly, our study relied on data from administrative registries, namely Statistisches Bundesamt and InEK, which may underestimate or overestimate the true incidence of rRD. It depends on reporting the correct diagnosis, which may be subject to different motivations. However, it is essential to know that reporting their correct data is legally mandatory for all hospitals in Germany; thus, the effect should be minor. Additionally, the lack of detailed information on potential risk factors, such as myopia, ocular trauma, and previous ocular surgeries, limited our ability to explore their impact on the incidence of rRD.

### Future directions

Future research should investigate the role of specific risk factors in the incidence of rRD. Longitudinal studies incorporating comprehensive data on myopia, ocular trauma, and previous ocular surgeries are needed to better understand their associations with rRD incidence. Moreover, exploring the underlying mechanisms behind sex disparities and differential annual per cent changes in incidence rates will contribute to a better understanding of the epidemiology of rRD.

## Conclusion

In conclusion, this study identified a significant and progressive increase in the incidence of rRD in Germany from 2005 to 2021. Males had a higher incidence rate than females, and the annual per cent change in incidence rates was more pronounced among males. Age also played a crucial role, with rRD primarily affecting individuals in their sixth decade of life. The rising incidence of rRD and its associated risk factors highlight the need for enhanced surveillance, early detection strategies, and targeted interventions to effectively manage this sight-threatening condition.

### Supplementary Information

Below is the link to the electronic supplementary material.

Supplementary file1 (DOCX 21.8 KB)

## Data Availability

Datasets related to this article can be requested from DESTATIS.DE and found at https://www.g-drg.de/inek-datenportal, an open-source online data repository hosted by Institut für das Entgeltsystem im Krankenhaus (InEK)/the Institute for the Remuneration System in Hospitals.
