# Supplementary material for: The rising tide of rhegmatogenous retinal detachment in Germany: a nationwide analysis of the incidence, from 2005 to 2021
Source: Graefes Arch Clin Exp Ophthalmol. 2024 Mar 11;262(8):2431–8. doi: 10.1007/s00417-024-06392-2 (PMC11271417; doi:10.1007/s00417-024-06392-2)
Supplement: Supplementary file 1 — Supplementary file1 (DOCX 21.8 KB) [file 417_2024_6392_MOESM1_ESM.docx]

Supplementary Table 1: Calculation of de novo Incidence Rate

|  | Actual Admissions | | | |  | Based on linearly estimated Re-operation rates | | | |
| --- | --- | --- | --- | --- | --- | --- | --- | --- | --- |
| Year | Total Admissions | Admissions /population  (Raw incidence) | Incidence LCI | Incidence UCI | Reoperations  (Modelled 2005-2018)  (Actual 2019-2021 / yellow-highlighted) | New Patients  (Without reoperations) | Incidence  (DeNovo Incidence) | Incidence - LCI | Incidence - UCI |
| 2005 | 13,906 | 16.9 | 16.6 | 17.2 | 1325 | 12,582 | 15.3 | 15.0 | 15.5 |
| 2006 | 14,728 | 17.9 | 17.6 | 18.2 | 1433 | 13,295 | 16.2 | 15.9 | 16.4 |
| 2007 | 15,269 | 18.6 | 18.3 | 18.9 | 1542 | 13,728 | 16.7 | 16.4 | 17.0 |
| 2008 | 15,945 | 19.4 | 19.1 | 19.7 | 1650 | 14,295 | 17.4 | 17.1 | 17.7 |
| 2009 | 17,152 | 21.0 | 20.7 | 21.3 | 1759 | 15,394 | 18.8 | 18.5 | 19.1 |
| 2010 | 17,604 | 21.5 | 21.2 | 21.8 | 1867 | 15,737 | 19.2 | 18.9 | 19.6 |
| 2011 | 18,483 | 23.0 | 22.7 | 23.4 | 1976 | 16,508 | 20.6 | 20.2 | 20.9 |
| 2012 | 19,311 | 24.0 | 23.7 | 24.3 | 2084 | 17,227 | 21.4 | 21.1 | 21.7 |
| 2013 | 19,610 | 24.3 | 23.9 | 24.6 | 2193 | 17,418 | 21.6 | 21.2 | 21.9 |
| 2014 | 19,885 | 24.5 | 24.1 | 24.8 | 2301 | 17,584 | 21.7 | 21.3 | 22.0 |
| 2015 | 21,121 | 25.7 | 25.4 | 26.1 | 2410 | 18,712 | 22.8 | 22.4 | 23.1 |
| 2016 | 21,970 | 26.6 | 26.3 | 27.0 | 2518 | 19,452 | 23.6 | 23.2 | 23.9 |
| 2017 | 22,922 | 27.7 | 27.3 | 28.0 | 2627 | 20,296 | 24.5 | 24.2 | 24.9 |
| 2018 | 24,396 | 29.4 | 29.0 | 29.7 | 2735 | 21,661 | 26.1 | 25.7 | 26.4 |
| 2019 | 23,955* | 28.8 | 28.4 | 29.2 | 2916 | 21,046 | 25.3 | 25.0 | 25.6 |
| 2020 | 22,355 | 26.9 | 26.5 | 27.2 | 2807 | 19,548 | 23.5 | 23.2 | 23.8 |
| 2021 | 24,045 | 28.5 | 28.1 | 28.9 | 3133 | 20,912 | 24.8 | 24.5 | 25.1 |

* We used a mean value between the InEK and DESTATIS, LCI: Lower 95% confidence Interval boundary, UCI: Upper 95% confidence interval boundary
